# Ulcerate colitis and protein C system: is there a link of causality

**DOI:** 10.1007/s00384-015-2453-x

**Published:** 2015-12-05

**Authors:** Xuhong Lin, Dandan Wei, Huichao Wang, Zhaoya Yu, Yuxia Li, Yanmin Li, Yanan Wen, Xuequn Ren, Guanchang Cheng

**Affiliations:** Department of Clinical Laboratory, Translational Medicine Center, Huaihe Hospital Affiliated to Henan University, 115 Ximen Street, Kaifeng City, Henan Province China; Department of Nephrology, First Affiliated Hospital of Henan University, 357 Ximen Street, Kaifeng City, Henan Province China; Department of General Surgery, Huaihe Hospital Affiliated to Henan University, 115 Ximen Street, Kaifeng City, Henan Province China; Department of Cardiovascular Medicine, Huaihe Hospital Affiliated to Henan University, 115 Ximen Street, Kaifeng City, Henan Province China

Dear Editor:

Ulcerate colitis (UC), the main type of inflammatory bowel disease (IBD), is an unexplained immune-mediated intestinal mucosal inflammation with higher cancer risk. Because of an interaction between inflammation and coagulation in the disease progression, hypercoagulative state is often seen in patients of UC. Endothelial cell damage plays an important role in the activation of coagulation system in patients with UC, while protein C (PC) system, a well-characterized anticoagulant system located on the vascular endothelial cell surface, which consists of protein S (PS), endothelial cell protein C receptor (EPCR), PC, protein C inhibitor, and thrombomodulin (TM), is a mediator of endothelial function. Recent studies indicated that PC system plays an important role in regulating intestinal homeostasis both in human intestinal microvascular endothelial cells and animal models of colitis. Therefore, we speculate that the imbalance of PC system may be one of the pathogenesis of UC patients. Our in vivo study verified that the degree of inflammation was negatively correlated with the PC activity, and mouse colonic macrophages and mucosa microvascular endothelial cells are suggested as the major mediators involved. Until today, it is unclear why PC system changed, and how macrophages and mucosa microvascular endothelial cells interact with each other in UC pathology, so it is important to investigate the changes and mechanisms of PC system, and to find new drug target. Further research in vitro revealed that TNF-α and IL-6 levels were increased in the supernatant of macrophages from dextran sulfate sodium (DSS) mice colonic tissue, and these conditions were only seen in CD14^+^CD64^+^ subtype macrophages. In addition, after incubation of TNF-α or IL-6 with colonic mucosal microvascular endothelial cells, the activity of PC system was inhibited, manifested as decreased APC activity, and downregulated expression of EPCR. Furthermore, β-arrestin-pJNK MAPK expressions were upregulated by real-time PCR and Western blot. Taken together into account, due to complex UC mechanism, its pathogenesis is unclear, and there is little research about colonic mucosal microvascular, especially the study for PC system changes in the pathogenesis of UC is rare. In our study, we found that in DSS-induced mouse colitis, colonic CD14^+^CD64^+^ macrophages produced more inflammatory cytokines, which result in inhibited PC pathway via mucosal microvascular endothelial cells, and β-arrestin-pJNK MAPK signal pathway may involved. Therefore, enhancing the activity of PC system provides a major theoretical and clinical significance to find new drug target for the treatment of UC.

